# Seasonal and daytime variation in multiple immune parameters in humans: Evidence from 329,261 participants of the UK Biobank cohort

**DOI:** 10.1016/j.isci.2021.102255

**Published:** 2021-03-01

**Authors:** Cathy Wyse, Grace O'Malley, Andrew N. Coogan, Sam McConkey, Daniel J. Smith

**Affiliations:** 1 School of Physiotherapy, Division of Population Health Sciences, Royal College of Surgeons in Ireland, Beaux Lane House, Mercer Street Lower, Dublin, Ireland; 2 Kathleen Lonsdale Institute for Human Health Research, Maynooth University, Maynooth, Kildare, Ireland; 3 Royal College of Surgeons in Ireland: University of Medicine and Health Science, Dublin, Ireland; 4 Institute of Health and Wellbeing, University of Glasgow, Glasgow, Scotland

**Keywords:** Immunology, Immune Response, Chronobiology

## Abstract

Seasonal disease outbreaks are perennial features of human infectious disease but the factors generating these patterns are unclear. Here we investigate seasonal and daytime variability in multiple immune parameters in 329,261 participants in UK Biobank and test for associations with a wide range of environmental and lifestyle factors, including changes in day length, outdoor temperature and vitamin D at the time the blood sample was collected. Seasonal patterns were evident in lymphocyte and neutrophil counts, and C-reactive protein CRP, but not monocytes, and these were independent of lifestyle, demographic, and environmental factors. All the immune parameters assessed demonstrated significant daytime variation that was independent of confounding factors. At a population level, human immune parameters vary across season and across time of day, independent of multiple confounding factors. Both season and time of day are fundamental dimensions of immune function that should be considered in all studies of immuno-prophylaxis and disease transmission.

## Highlights

• Seasonal outbreaks are common features of human infectious disease
• Immune parameters varied across seasons and time of day in UK Biobank participants
• Innate variability in immune parameters could influence human susceptibility to disease

## Graphical Abstract

**Figure:**
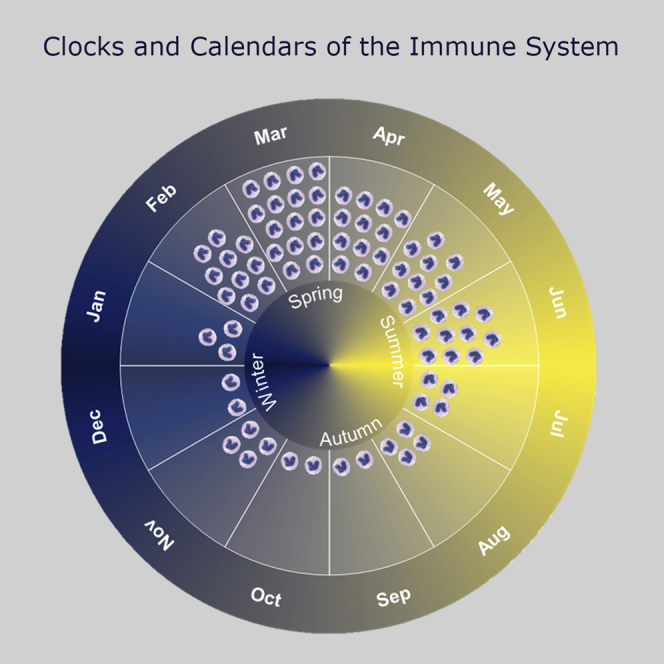

## Introduction

Annual cycles in vulnerability to infectious disease are an established feature of human epidemiology: most respiratory viruses cause winter-time infection and polio is principally a summer-time disease (Dowell and Shang Ho, 2004). Childhood infectious diseases (meningitis, mumps, pertussis, and varicella) (Shah et al., 2006), and many of the contagious diseases that affect domestic animals (Poljak et al., 2014) (Jactel et al., 1990) are seasonal as are relapses in autoimmune diseases (Harding et al., 2017; Mori et al., 2019). The factors that mediate this seasonality are poorly understood and circannual patterns are simply an assumed component of the dynamics of infectious diseases. In addition to seasonality, animals and humans are more susceptible to infectious disease during the resting phase of their daily cycle (Tognini et al., 2017), adding a further circadian dimension to disease vulnerability.

The axial and orbital rotations of the Earth generate predictable seasonal and daily rhythms of light and darkness. These conditions in turn generate circadian and seasonal oscillations in ambient temperature, food availability, predation, and risk of infection. Evolution has equipped animals with innate timing mechanisms, or “clocks”, that synchronize physiology to these recurring periods of increased risk. The circadian clock is generated by a series of interconnected transcription–translation feedback loops that regulate the expression of a panel of clock-controlled genes (Takahashi, 2017). Most mammalian cells contain a molecular clock and overall rhythmicity is maintained by a master clock located in the suprachiasmatic nuclei of the hypothalamus, conferring time dependence on most physiological parameters through hormonal and neural signals (Takahashi, 2017). Mice deficient in the cryptochrome clock genes (*Cry1* and *Cry2*), show elevated proinflammatory cytokines (Narasimamurthy et al., 2012), and an autoimmune phenotype (Cao et al., 2017), while loss of clock function by deletion of the clock gene *Bmal1* was associated with augmented immunity against bacterial infection (Kitchen et al., 2020), all evidence for a close association between circadian timing mechanisms and immune function.

The mechanisms driving seasonality in humans are unclear, but in animals, the seasonal clock is generated by changes in thyroid hormones in the brain that respond to day length signaled by the pineal hormone melatonin (Wood and Loudon, 2014). The circadian clock is entrained by the 24-hr photoperiod, while the seasonal clock entrains to day length patterns in Northern latitudes and to seasonal patterns in rain and food availability in tropical regions, where day length is constant (Bronson, 2009; Stevenson and Prendergast, 2015). This is analogous with aspects of seasonality of the human immune system, where viral infection and immune cell numbers are associated with day length (e.g. winter peak in influenza) in Northern clines and with climatic changes in tropical regions (Tamerius et al., 2011).

Together, the seasonal and circadian clock synchronize physiology in two dimensions of time, optimizing homeostasis by anticipating changes in the environment. For example, plants (Gangappa and Kumar, 2018), fish (Fortes-Silva et al., 2019), birds (Markowska et al., 2017) and mammals (Pawlak et al., 2005; Yellon and Tran, 2002) all align their immune defense with the time of day that pathogenic and physical challenge are most likely. This conservation across the biological kingdoms is strong evidence that temporal modulation of immune function is an ancient and fundamental mechanism that has evolved to optimize survival in variable environmental conditions.

Laboratory experiments corroborate epidemiological evidence of circadian and seasonal rhythms in disease susceptibility. For example, mice are more resilient to experimental inflammatory (Feigin et al., 1969; Halberg et al., 1960), infectious (Bellet et al., 2013; Edgar et al., 2016; Wongwiwat et al., 1972) and physical challenges (Cable et al., 2017) delivered at night (their active circadian phase) or in summer (Schulman and Kilbourne, 1963). Importantly, these daily cycles in vulnerability persist in constant conditions (photoperiod, temperature, or humidity) (Schulman and Kilbourne, 1963) (Shackelford and Feigin, 1973) and are absent in animals lacking a circadian clock (Curtis et al., 2015; Edgar et al., 2016), demonstrating clock-mediated regulation that is not driven by current environmental conditions. Similar to rodents, humans are more resistant to the effects of inflammatory or infectious challenge (Alamili et al., 2014) (Pollmächer et al., 1996) delivered during their active circadian phase (day time), or in summer (W M Lee et al., 2012) (Shadrin et al., 1977; Zykov and Sosunov, 1987).

Circulating white blood cell counts are known to oscillate across 24hr under basal conditions, reflecting distribution of cells between tissues and the periphery (Stenzinger et al., 2019). Importantly, these rhythms persist in constant conditions and are absent in animals with ablated clock function (Stenzinger et al., 2019), indicating that they are mediated via innate circadian timing mechanisms. The extensive data collection within UK Biobank represents an unprecedented opportunity to assess seasonal and time-of-day variation in levels of human immune parameters. Here, we provide evidence of endogenous seasonal and daytime variability in human immune function at a population level, and we demonstrate that these patterns are independent of a wide range of demographic, environmental, and lifestyle factors.

## Results

The exclusion criteria for this study resulted in the removal of 173,275 study participants. The remaining cohort was mostly White (98%), with the other ethnic groups poorly represented (<2% participants). Summary data that describes the demography and lifestyle of the 329,261 participants that were eligible for inclusion are given in Table 1.

**Table 1:** Demographic and lifestyle characteristics

Variable	(n = 329,261)
Age (years)	
Mean (SD)	55.8 (8.19)
Townsend deprivation index	
Mean (SD)	−1.55 (2.94)
Ethnicity	
White	306146 (91.0%)
Physical activity	
Mean (SD)	46.0 (62.8)
Sedentary behavior	
Mean (SD)	4.90 (2.24)
BMI	
Mean (SD)	26.8 (4.34)
Smoking	
Yes	31,063 (9.2%)
Chronotype	
More evening than morning	81,240 (24.1%)
Evening	23,185 (6.9%)
More morning than evening	105763 (31.4%)
Morning	78,226 (23.2%)

**Figure 1:**
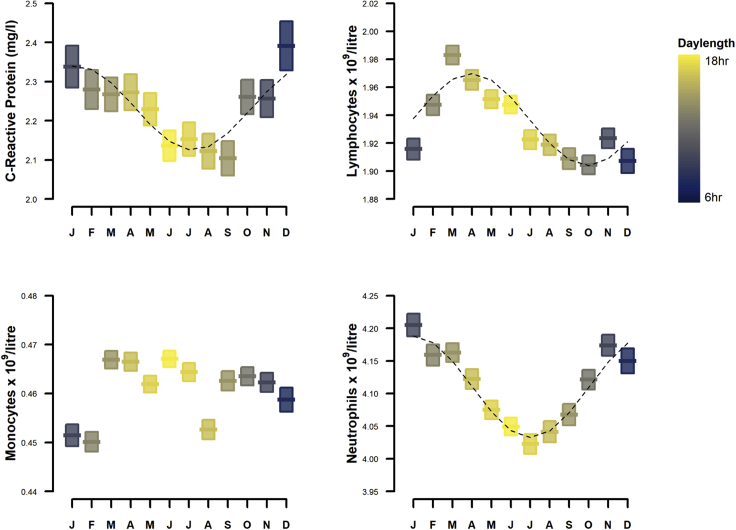
Annual variation in total monocytes, neutrophils, lymphocytes, and CRP. Data are mean (bars) and 95% confidence intervals (boxes), with fitted cosinor curves (dotted line). Daylength is indicated by the box color gradients

**Figure 2:**
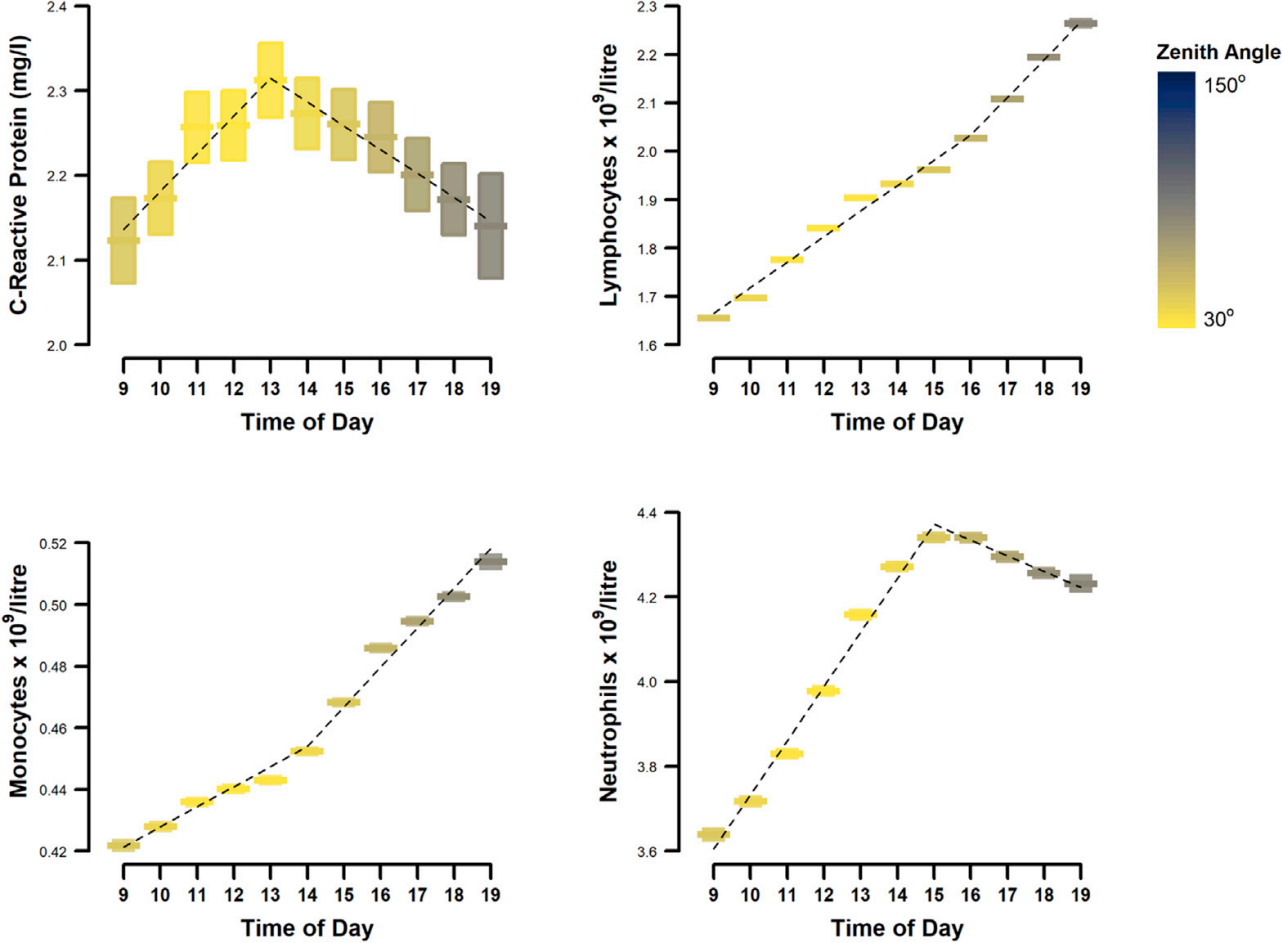
Daytime variation in monocytes, neutrophils, lymphocytes, and CRP. Data are mean (bars) and 95% confidence intervals (boxes), with fitted segmented regression lines (dotted black lines). The color gradient represents mean zenith angle of the sun at each time point is given to indicate daylight

Mean values for WBC percentage and CRP were plotted against month and time of day for all participants, and annual and daily variation was evident on visual inspection and univariate analysis (Figures 1 and 2), but there was no seasonal or diurnal variation in the titer levels of any antigen (data not shown. The probability of seropositive status to any of the 20 antigens analysis was not associated with the month or time of day of analysis (data not shown). Summary data for white blood cell count, vitamin D, and CRP levels at all time points are shown in supplementary information (Tables S1–S4). Mean and 95% confidence intervals for monthly data with fitted cosinor models for lymphocyte, monocyte, neutrophil, and CRP are shown in Figures 1 and 2. Cosinor analysis showed that the seasonal patterns were statistically significant for a 12-month assumed periodicity for CRP and WBC counts (Table 2).

**Table 2:** Parameters describing the amplitude, annual peak (acrophase), and mean value (mesor) predicted by fitted cosinor model

Day length	Amplitude	Acrophase	Bathyphase	Mesor	p
**Neutrophils** (10^9^/L)	0.80	Jan	Jul	4.10	<0.001
**Lymphocytes** (10^9^/L)	0.03	April	Oct	1.93	<0.001
**CRP** (mg/L)	0.10	Jan	Jun	2.23	<0.001

Data are mean and sd.

CRP levels were higher in the winter months, peaking in December, with lowest levels in July. The seasonal pattern of neutrophil counts was similarly higher in winter, peaking in Jan with lowest levels recorded in summer (July). The seasonal pattern of lymphocyte counts peaked in spring (March) and troughed in autumn (October) (; Figure 1). There was no significant seasonal pattern in monocyte counts.

Multiple linear regression was next applied to investigate whether the immune parameters were associated with day length and, if so, whether these were independent of other lifestyle and environmental factors that could confound associations via unrelated seasonality. There was some degree of correlation between outdoor temperature and day length, as expected, but the multicollinearity diagnostic indices, VIF (variance inflation factor) and tolerance did not reach the thresholds (>10 and <0.2, respectively) to indicate problematic multicollinearity (Table S5). CRP levels, as well as neutrophil and lymphocyte counts were found to be significantly associated with day length, independent of demographic, lifestyle, and environmental factors (Table 3) including outdoor temperature and vitamin D. The relationship between vitamin D and CRP was found to be dependent on BMI, and an interaction term to account for this effect was included in the CRP regression model. Interaction was also detected between vitamin D and sex for all WBC markers, and these interaction terms were added to the regression models (supplementary information Tables S5 and S6). In the fully adjusted model, neutrophil count and CRP showed significant negative associations with day length, while lymphocyte count was positively associated, as also shown in cosinor analysis. Monocyte count was not significantly associated with day length in the fully adjusted model (Model 3).

**Table 3:** Associations between day length and CRP, lymphocyte, neutrophil, and monocyte count

Day length	Model 1		Model 2		Model 3	
White blood cells (10^9^/L)	−0.011^∗∗∗^	[-0.013,-0.009]	−0.008^∗∗∗^	[-0.011,-0.006]	−0.011^∗∗∗^	[-0.015,-0.007]
Neutrophils (10^9^/L)	−0.015^∗∗∗^	[-0.017,-0.014]	−0.013^∗∗∗^	[-0.015,-0.011]	−0.014^∗∗∗^	[-0.018,-0.011]
Monocytes (10^9^/L)	0.001^∗∗∗^	[0.001,0.001]	0.001^∗∗∗^	[0.001,0.001]	−0.000	[-0.001,-0.000]
Lymphocytes (10^9^/L)	0.002^∗∗∗^	[0.002,0.003]	0.003^∗∗∗^	[0.002,0.004]	0.004^∗∗∗^	[0.003,0.005]
CRP (mg/L)	−0.006^∗∗∗^	[-0.007,-0.004]	−0.005^∗∗∗^	[-0.006,-0.003]	−0.004^∗∗∗^	[-0.007,-0.002]

Data are expressed as regression coefficients (B), with 95% confidence intervals in parentheses.

Model 1 was adjusted for age, sex, ethnicity, deprivation.

Model 2 was adjusted for Model 1 + BMI, physical activity, sedentary behavior, sleep duration, chronotype, shiftwork, smoking, alcohol.

Model 3 was adjusted for Model 2 + vitamin D, outdoor temperature, time of day, blood analyzer, and UK Biobank assessment center.

Segmented linear regression analysis of CRP and WBC counts over the daily time course showed significant daytime variation that was represented by segmental regression lines (Figure 2). The peaks and trough (break points) for each marker are shown in Table 4. All white blood cells showed significant daily variation, with counts lowest in the early morning and increasing as the day progressed. Neutrophil count reached a plateau at 3pm. CRP levels were highly variable, peaking at 1pm, and decreasing thereafter (Table 4).

**Table 4:** Segmented regression parameters showing predicted break points for each segment and regression coefficient for overall segmented linear model

Time of day	Break point time of day (se)	p∗
White blood cells (10^9^/L)	14.34 (0.069)	<0.001
Neutrophils (10^9^/L)	14.62 (0.040)	<0.001
Monocytes (10^9^/L)	13.27 (0.160)	<0.001
Lymphocytes (10^9^/L)	16.12 (0.180)	<0.001
CRP (mg/L)	12.71 (0.220)	<0.01

∗Davies' test was applied to test for significant differences of slopes between each segmented relationship.

Linear regression analysis demonstrated that the daytime changes in WBCs and CRP were in most cases independent of lifestyle and environmental factors (Table 5). The morning ascending segment of the CRP daily curve was the only section of any of the curves that did not retain statistical significance after adjustment. However the daily CRP curve showed a significant relationship with time of day for the later parts of the day, after the break point at 1pm.

**Table 5:** Associations between time of day and CRP, lymphocyte, neutrophil, and monocyte counts

Day length	Model 1		Model 2		Model 3	
WBCs (10^9^/L)						
Segment 1	0.207^∗∗∗^	[0.203,0.211]	0.199^∗∗∗^	[0.195,0.203]	0.197^∗∗∗^	[0.193,0.202]
Segment 2	0.078^∗∗∗^	[0.073,0.082]	0.082^∗∗∗^	[0.078,0.086]	0.082^∗∗∗^	[0.078,0.087]
Neutrophils (10^9^/L)						
Segment 1	0.094^∗∗^	[0.033,0.156]	0.095^∗∗^	[0.030,0.159]	0.101^∗∗^	[0.036,0.165]
Segment 2	0.268^∗∗∗^	[0.243,0.293]	0.262^∗∗∗^	[0.236,0.289]	0.259^∗∗∗^	[0.233,0.285]
Monocytes (10^9^/L)						
Segment 1	0.085^∗∗∗^	[0.048,0.122]	0.086^∗∗∗^	[0.047,0.125]	0.085^∗∗∗^	[0.046,0.124]
Segment 2	−0.206^∗∗∗^	[-0.235,-0.177]	−0.199^∗∗∗^	[-0.230,-0.168]	−0.195^∗∗∗^	[-0.227,-0.164]
Lymphocytes (10^9^/L)						
Segment 1	0.051^∗∗∗^	[0.050,0.052]	0.050^∗∗∗^	[0.049,0.051]	0.050^∗∗∗^	[0.049,0.051]
Segment 2	0.082^∗∗∗^	[0.079,0.085]	0.085^∗∗∗^	[0.082,0.088]	0.085^∗∗∗^	[0.082,0.088]
CRP (mg/L)						
Segment 1	0.015	[-0.000,0.030]	0.009	[-0.007,0.025]	0.010	[-0.006,0.026]
Segment 2	−0.009^∗^	[-0.017,-0.001]	−0.013^∗∗∗^	[-0.021,-0.006]	−0.013^∗∗^	[-0.021,-0.005]

Time of day is represented by 2 linear splines, to account for non-linear relationships with the independent variables.

Data are expressed as regression coefficients (B), with 95% confidence intervals in parentheses.

Model 1 was adjusted for age, sex, ethnicity, deprivation.

Model 2 was adjusted for BMI, physical activity, sedentary behavior, sleep duration, chronotype, smoking, alcohol.

Model 3 was adjusted for Model 2 + day length, blood analyzer and UK Biobank assessment center.

## Discussion

The human immune system is not constant over 24hr or across the seasons and the time of exposure to pathogens is an important consideration in determining risk of infection Here we report seasonal and daytime patterns in immune cells and inflammatory markers within a large sample of the UK population. Importantly, we demonstrate that these patterns are independent of multiple demographic, lifestyle and local environmental variables, supporting the existence of endogenous seasonal and daytime patterns in human immune parameters. These findings highlight the importance of future studies to understand the time dimensions of immune function and their implications for preventing and controlling outbreaks of infectious disease.

UK Biobank combines multiple blood, demographic, and environmental parameters in a large sample of the UK population that allowed us to generate novel information on the factors that mediate daytime and seasonal variations in peripheral immune cells. It was possible to adjust for multiple lifestyle-associated parameters that might be seasonal such as physical activity and sleep duration, and unlike previous studies, we were also able to adjust for the effects of vitamin-D and outdoor temperature. The timing of sample collection was randomized in this study, which allowed us to exclude the possibility of bias through self-selection of the time of sampling. Finally, this study contributes to current knowledge by demonstrating significant daytime and seasonal variability in immune cells in the biggest population sample reported to date.

The greatest seasonal and daytime changes in this study were seen in lymphocyte numbers, with high-amplitude variation over seasons and days. Lymphocytes were lower during the early parts of the day, increasing as the day progressed, consistent with previous reports that lymphocytes circulating in blood are lower during the respective active phase of humans (Ackermann et al., 2012) (He et al., 2018) (Lange et al., 2010) (Born et al., 1997) (Abo et al., 1981) and rodents (Druzd et al., 2017; He et al., 2018) (Suzuki et al., 2016). Interestingly, longer sleep times were associated with numbers of circulating white blood cells across multiple mammalian species (Preston et al., 2009), further supporting a relationship between the timing of activity and immune function. Circadian rhythms in the homing and egress of lymphocytes through the lymphatic system and other tissues underlies these diurnal changes of lymphocyte numbers in blood (Druzd et al., 2017; He et al., 2018) (Pick et al., 2019). Since the lymph nodes contain the interaction between lymphocytes and antigen, longer accumulation times increase antigen encounters and potentiate the adaptive immune response (Scheiermann et al., 2018) (Suzuki et al., 2016). Consequently, lymphocyte numbers in the periphery drop as trafficking to the tissues increases, along with increased tissue surveillance and resistance to infection (He et al., 2018) (Pick et al., 2019). Circadian rhythms of lymphocyte trafficking to the periphery are abolished by genetic ablation of clock function and persist in constant conditions (Deprés-Brummer et al., 1997; Druzd et al., 2017), confirming their regulation by the innate circadian clock in mice. These endogenous rhythms are associated with time-of-day dependent changes in adaptive immunity, including amplified response to induction of autoimmunity (experimental autoimmune encephalomyelitis), immuniZation (Suzuki et al., 2016) and viral infection (influenza) during the active phase (Druzd et al., 2017). Thus the time of day that pathogenic challenge occurs affects the adaptive immune response generated days later, one of the mechanisms through which lymphocyte trafficking might modulate seasonal and circadian vulnerability to infection. In agreement with previous findings (Aguirre-Gamboa et al., 2016; Liu and Taioli, 2015), lymphocytes were positively associated with day length in our study; cell numbers were lower in autumn and peaked in spring. These seasonal variations in peripheral lymphocyte counts suggest that humans have some capacity for seasonal regulation of lymphocyte trafficking that could contribute to seasonality in susceptibility to infection.

Previous studies have reported seasonal and circadian patterns in antibody titers in humans (Rosenblatt et al., 1982), IgM (Dozier et al., 1997), often discovered serendipitously in the course of other investigations (Dozier et al., 1997). Leukocytes collected at different times of year showed decreased *ex vivo* response (thymidine incorporation, cytokine release) to activation in winter time in humans and rats (Boctor et al., 1989; Brock, 1983, 1987) (Amat and Torres, 1993), and diurnal patterns in antibody titers (Kurupati et al., 2017) and in *ex vivo* response to stimulation of PBMCs (Van Rood et al., 1991) (de Bree et al., 2020) have been reported in humans. This study in UK Biobank is the first to investigate circadian and seasonal patterns in antibody titers to common infectious agents at a population level. Despite our comparatively large sample size, we found no evidence for seasonal or daily variation in antibody titers or in the probability of testing immunopositive to any of the 20 antigens investigated in this study. However, the antibody response to vaccination or viral infection and subsequent decay is subject to wide variation between individuals (Antia et al., 2018), which is not accounted for by the cross-sectional design of the present study. Longitudinal experiments are required to establish if antibody titers vary by season or time of day and how this might impact on response to vaccination or infection. Daytime variation in antibody titers could confound studies of the efficacy of vaccination that use antibody response as an outcome variable (Kurupati et al., 2017) (Long et al., 2016a, 2016b), and future investigations within individuals and with multiple sample time points are warranted to understand basal variation in antibody titers.

Blood neutrophil counts were lowest in early morning in the UK Biobank participants, increasing thereafter to plateau after 3pm. Previous studies demonstrated comparable circadian rhythms in peripheral neutrophil counts that were low in the rest phase, and that increased over the active phase in both humans and mice (Ackermann et al., 2012)(Jilma et al., 1999). Neutrophils have a half-life less than 24hr, and circadian rhythmicity is regulated through clock-controlled oscillations in chemokine pathways that drive release of young cells in the active phase and clearance of aged neutrophils from the periphery in the resting phase (Adrover et al., 2019; Casanova-Acebes et al., 2013). These rhythms in neutrophil tissue migration were shown to underlie increased resistance to infection (*Candidia albicans*), during the active phase in mice (Adrover et al., 2019) and to diurnal variation in bactericidal function *ex vivo* in human neutrophils (Ella et al., 2016). Neutrophil counts were negatively associated with day length in our study, in agreement with previous studies in humans living at temperate latitudes (Goldinger et al., 2015; Liu and Taioli, 2015). We extend these findings to demonstrate high peripheral neutrophil counts in winter time at a population level that were related to annual photoperiod, independent of participant lifestyle, local environmental conditions and vitamin D. In addition to total counts, previous studies have demonstrated seasonality of functional aspects of neutrophil immune function, including adhesive capacity, CD11b/CD18 expression and ROS production, resulting in augmented bactericidal properties of neutrophils collected in summer (Klink et al., 2012). The seasonal and daytime patterns in neutrophil count reported here, and in previous studies support evidence from animal studies that time-dependent cycles of tissue migration could contribute to neutrophil-mediated resilience to infection during the active phase, and relative vulnerability to infection in winter time (Adrover et al., 2019; Casanova-Acebes et al., 2013).

Monocyte counts were lower in the morning compared to evening in UK Biobank participants, consistent with previous reports that monocytes increase during the active phase in mice (Nguyen et al., 2013) and humans (Born et al., 1997). The acute phase protein, CRP showed a weak daily pattern in this study, with levels higher in daytime, again corroborating previous reports of diurnal patterns of CRP in humans (Morris et al., 2017) (Chiriboga et al., 2009; Liu and Taioli, 2015; Rudnicka et al., 2007) The immune function of tissue macrophages was shown to be regulated by intrinsic circadian timing mechanisms that were independent of systemic glucocorticoid secretion (Keller et al., 2009). Circadian rhythms in the circulation and tissue migration of monocytes in mice were similarly regulated through an innate cell-intrinsic clock mechanism and their oscillation coincides with an enhanced inflammatory response when monocytes are decreasing at the beginning of the rest phase (Nguyen et al., 2013) and increased lethality of endotoxic challenge at this time (Halberg et al., 1960). In agreement, human volunteers show a heightened response to endotoxic challenge in the evening (Alamili et al., 2014; Pollmächer et al., 1996). This increased inflammatory response in the active phase might maximize innate immune defense at a time when pathogenic challenge is most likely (Curtis et al., 2014) but could also leave animals more vulnerable to the toxic effects of augmented inflammation.

Monocyte counts are higher in winter in some (Aguirre-Gamboa et al., 2016) but not all (Liu and Taioli, 2015) previous studies. There was no evidence of a seasonal pattern in UK Biobank participants, and monocytes were not associated with day length in the fully adjusted model. While peripheral counts are not always seasonal, monocyte function shows strong seasonality *ex vivo*, with an augmented proinflammatory response to activation in summer time (Myrianthefs et al., 2003; ter Horst et al., 2016). We found a weak seasonal pattern in the acute phase protein, CRP in UK Biobank, with levels higher in the winter months. Peripheral CRP and other proinflammatory markers were higher in winter in many studies in humans (Sung, 2006) (Liu and Taioli, 2015), and this is thought to contribute to seasonal prevalence of cardiovascular disease (Sartini et al., 2017).

Seasonality of human viral infections is generally and intuitively thought to be driven by annual changes in temperature or humidity, but there is increasing evidence that innate variation in host disease susceptibility is an important contributor. In support of this, many diseases are seasonal in tropical regions where temperature and humidity are constant (Bloom-Feshbach et al., 2013) (Tamerius et al., 2011). Furthermore, outbreaks of influenza occur annually and simultaneously at latitudes that are oceans apart (Lofgren et al., 2007) despite variations in local climatic conditions and human behavior. Recurrent seasonality is a feature of the epidemiology of infectious disease in animals that do not share human winter time behaviors such as increased time indoors, crowding or school terms. The prevalence of human respiratory viruses does not correspond with the prevalence of the respiratory disease they cause; remarkably, detection of viral infection is relatively low in the months that respiratory disease is highest (Lee et al., 2012).

Vitamin D is suspected to contribute to disease seasonality due to known associations with immune function and highly seasonal serum levels (Cannell et al., 2006), but this postulation is not corroborated by models that compared serum vitamin D with influenza transmission in population based studies (Shaman et al., 2011; Xu et al., 2016). Our findings in UK Biobank showed that seasonal changes in white blood cells and CRP were related to day length independent of vitamin D levels, in agreement with evidence that circulating vitamin D was not responsible for seasonality in the proinflammatory functions of human monocytes (ter Horst et al., 2016). Finally, evidence of widespread seasonal regulation of transcription of genes regulating immune function and of reversed expression patterns in Northern and Southern hemispheres strongly supports endogenous regulation of seasonality in human immune function (Dopico et al., 2015). Seasonality of human infectious disease may be driven by an endogenous circannual rhythmicity in host immunity that generates cycles of enhancement and suppression of immune function and windows of vulnerability to infection, as proposed by Dowell in 2001 (Dowell, 2001).

### Limitations of the study

This study in UK Biobank is the largest investigation of the seasonal and daytime patterns in human immune cells, inflammatory markers and antibody titers at population level, but our results are subject to many important limitations. Although UK Biobank represents a very large population sample of middle-aged UK adults, only 5.5% of those who received an invitation volunteered to take part. A broad range of socioeconomic groups are represented but ethnic diversity is low (Sudlow et al., 2015). Furthermore, UK Biobank specifically recruited participants aged 40–69 years, so our findings may not apply to younger people. Some of the data that we analyzed were self-reported, including ethnicity, physical activity, health status and chronotype, and misclassification errors are possible. The participants denied chronic disease, but we cannot exclude the presence of acute infection at the time of assessment. The study design was cross-sectional, and a single blood sample was available from each participant so the influence of within subject variation cannot be assessed. We were unable to assess circadian patterns since there were no nighttime blood sample collections, and our results are limited to analysis of daytime variation. Nevertheless, the daily patterns we report in over 300,000 participants are consistent with the results of previous studies where blood was withdrawn at regular time points over 24hr under experimental conditions. We present results of total cell counts only, and further studies are required to investigate subtypes of lymphocytes and neutrophils. The immune parameters that we report are affected by a multitude of factors related to interactions between host, pathogen, and the environment. It is not possible to consider all of these in a population-based study, and the mechanisms driving the associations with day length and time of day that we report require investigation under controlled experimental conditions. Furthermore, the effect sizes we report are small, and likely to be of clinical significance for population-level disease control, rather than for the health of individuals. The strengths of this study are the large sample size and that the times of sample collection were randomly allocated to each participant. It is also a strength that we were able to investigate the effects of day length and time of day on immune parameters while adjusting for other factors thought to affect seasonal and daytime variability including physical activity, vitamin D, and outdoor temperature.

Seasonality in the epidemiology of infectious disease is considered to be generated by environment and pathogen related factors, and innate variability in host susceptibility to infection is rarely considered. Our findings of seasonal and daytime variability in multiple immune parameters in a large sample of the UK population under basal, free living conditions that were independent of environmental conditions, support the contribution of innate mechanisms to variability in disease susceptibility.

Future research should focus on whether elective restriction of human activity at times of increased vulnerability to infection through night time and winter curfews could control the spread of infectious disease by minimizing exposure to pathogens during susceptible periods. This is exactly the function that has driven the evolution of temporal regulation of the immune system and harnessing this innate attribute could optimize our resilience to COVID-19 and future pandemics.

### Resource availability

#### Lead contact

Further information and requests for resources and reagents should be directed to and will be fulfilled by the lead contact, Cathy Wyse (cathywyse@rcsi.com).

#### Materials availability

This study did not generate any new unique reagents.

#### Data and code availability

The data sets generated during this study are available at the UK Biobank repository, www.ukbiobank.ac.uk.

## Methods

All methods can be found in the accompanying Transparent Methods supplemental file.

## Supplemental information

Document S1. Transparent methods and Tables S1–S9

## Footnotes

Supplemental information can be found online at https://doi.org/10.1016/j.isci.2021.102255.
