# Supplementary material for: Seasonal and daytime variation in multiple immune parameters in humans: Evidence from 329,261 participants of the UK Biobank cohort
Source: iScience. 2021 Mar 1;24(4):102255. doi: 10.1016/j.isci.2021.102255 (PMC8010467; doi:10.1016/j.isci.2021.102255)
Supplement: Document S1. Transparent methods and Tables S1–S9 [file mmc1.pdf]

iScience, Volume *24*

Supplemental information

Seasonal and daytime variation in multiple immune parameters in humans: Evidence from 329,261 participants of the UK Biobank cohort

Cathy Wyse, Grace O'Malley, Andrew N. Coogan, Sam McConkey, and Daniel J. Smith

Table S1 Summary descriptive data for CRP, total white blood cells, monocytes, lymphocytes and neutrophils by month, mean [95% CI]. Related to Figure 1

**Month**	**Jan (N=24283)**	**Feb (N=26731)**	**Mar (N=32217)**	**Apr (N=28549)**	**May (N=33871)**	**Jun (N=33883)**	**Jul (N=27606)**	**Aug (N=24426)**	**Sep (N=23422)**	**Oct (N=27710)**	**Nov (N=26992)**	**Dec (N=18161)**
**CRP**												
mg/litre	2.337 (± 3.994)	2.279 (± 3.946)	2.264 (± 3.797)	2.266 (± 3.822)	2.232 (± 3.737)	2.133 (± 3.488)	2.152 (± 3.519)	2.122 (± 3.474)	2.101 (± 3.343)	2.260 (± 3.635)	2.257 (± 3.841)	2.392 (± 4.184)
**WBC**												
(10^9/litre)	6.792 (± 1.692)	6.769 (± 1.690)	6.831 (± 1.683)	6.772 (± 1.651)	6.708 (± 1.651)	6.690 (± 1.632)	6.630 (± 1.640)	6.627 (± 1.628)	6.650 (± 1.622)	6.699 (± 1.627)	6.775 (± 1.678)	6.725 (± 1.659)
**Neutrophils**												
(10^9/litre)	4.203 (± 1.324)	4.156 (± 1.315)	4.160 (± 1.294)	4.118 (± 1.275)	4.075 (± 1.279)	4.048 (± 1.254)	4.021 (± 1.267)	4.039 (± 1.254)	4.065 (± 1.255)	4.119 (± 1.279)	4.172 (± 1.305)	4.149 (± 1.300)
**Monocytes**												
(10^9/litre)	0.4514 (± 0.1679)	0.4499 (± 0.1655)	0.4668 (± 0.1592)	0.4665 (± 0.1568)	0.4626 (± 0.1562)	0.4675 (± 0.1554)	0.4644 (± 0.1535)	0.4524 (± 0.1540)	0.4624 (± 0.1524)	0.4633 (± 0.1544)	0.4621 (± 0.1594)	0.4586 (± 0.1669)
**Lymphocytes**												
(10^9/litre)	1.915 (± 0.5728)	1.946 (± 0.5948)	1.984 (± 0.6039)	1.966 (± 0.5856)	1.956 (± 0.5887)	1.950 (± 0.5764)	1.922 (± 0.5685)	1.918 (± 0.5732)	1.908 (± 0.5657)	1.903 (± 0.5618)	1.922 (± 0.5731)	1.906 (± 0.5822)

Table S2 Summary descriptive data for CRP, total white blood cells, monocytes, lymphocytes and neutrophils by time of day (hour), mean [95% CI]. Related to Figure 2

Time of Day	9 (N=20091)	10 (N=28642)	11 (N=34487)	12 (N=31376)	13 (N=31656)	14 (N=34257)	15 (N=32769)	16 (N=34022)	17 (N=31456)	18 (N=31409)	19 (N=13616)
**CRP**											
mg/litre	2.123 (± 3.538)	2.173 (± 3.597)	2.257 (± 3.838)	2.259 (± 3.614)	2.312 (± 3.853)	2.273 (± 3.826)	2.260 (± 3.725)	2.245 (± 3.772)	2.201 (± 3.778)	2.172 (± 3.700)	2.140 (± 3.535)
**WBC**											
(10^9/litre)	5.926 (± 1.514)	6.046 (± 1.546)	6.243 (± 1.564)	6.466 (± 1.579)	6.713 (± 1.616)	6.876 (± 1.622)	6.991 (± 1.608)	7.078 (± 1.635)	7.130 (± 1.616)	7.195 (± 1.624)	7.254 (± 1.639)
**Neutrophils**											
(10^9/litre)	3.640 (± 1.210)	3.717 (± 1.238)	3.829 (± 1.241)	3.978 (± 1.253)	4.159 (± 1.283)	4.271 (± 1.279)	4.340 (± 1.279)	4.339 (± 1.282)	4.295 (± 1.258)	4.256 (± 1.258)	4.231 (± 1.269)
**Monocytes**											
(10^9/litre)	0.4217 (± 0.1494)	0.4280 (± 0.1516)	0.4359 (± 0.1538)	0.4401 (± 0.1534)	0.4429 (± 0.1543)	0.4523 (± 0.1547)	0.4682 (± 0.1568)	0.4858 (± 0.1601)	0.4946 (± 0.1578)	0.5026 (± 0.1591)	0.5139 (± 0.1609)
**Lymphocytes**											
(10^9/litre)	1.654 (± 0.4742)	1.697 (± 0.4916)	1.776 (± 0.5167)	1.841 (± 0.5351)	1.904 (± 0.5525)	1.934 (± 0.5659)	1.963 (± 0.5606)	2.027 (± 0.5777)	2.107 (± 0.5852)	2.195 (± 0.6023)	2.263 (± 0.6292)

Table S3 Summary descriptive data for CRP, total white blood cells, monocytes, lymphocytes and neutrophils by month, median (interquartile range). Related to Figure 1

Month	Jan (N=24283)	Feb (N=26731)	Mar (N=32217)	Apr (N=28549)	May (N=33871)	Jun (N=33883)	Jul (N=27606)	Aug (N=24426)	Sep (N=23422)	Oct (N=27710)	Nov (N=26992)	Dec (N=18161)
**CRP**												
mg/litre	2.337 (3.994)	2.279 (3.946)	2.264 (3.797)	2.266 (3.822)	2.232 (3.737)	2.133 (3.488)	2.152 (3.519)	2.122 (3.474)	2.101 (3.343)	2.260 (3.635)	2.257 (3.841)	2.392 (4.184)
**WBC**												
(10^9/litre)	6.792 (1.692)	6.769 (1.690)	6.831 (1.683)	6.772 (1.651)	6.708 (1.651)	6.690 (1.632)	6.630 (1.640)	6.627 (1.628)	6.650 (1.622)	6.699 (1.627)	6.775 (1.678)	6.725 (1.659)
**Neutrophils**												
(10^9/litre)	4.203 (1.324)	4.156 (1.315)	4.160 (1.294)	4.118 (1.275)	4.075 (1.279)	4.048 (1.254)	4.021 (1.267)	4.039 (1.254)	4.065 (1.255)	4.119 (1.279)	4.172 (1.305)	4.149 (1.300)
**Monocytes**												
(10^9/litre)	0.4514 (0.1679)	0.4499 (0.1655)	0.4668 (0.1592)	0.4665 (0.1568)	0.4626 (0.1562)	0.4675 (0.1554)	0.4644 (0.1535)	0.4524 (0.1540)	0.4624 (0.1524)	0.4633 (0.1544)	0.4621 (0.1594)	0.4586 (0.1669)
**Lymphocytes**												
(10^9/litre)	1.915 (0.5728)	1.946 (0.5948)	1.984 (0.6039)	1.966 (0.5856)	1.956 (0.5887)	1.950 (0.5764)	1.922 (0.5685)	1.918 (0.5732)	1.908 (0.5657)	1.903 (0.5618)	1.922 (0.5731)	1.906 (0.5822)

Table S4 Summary descriptive data for CRP, total white blood cells, monocytes, lymphocytes and neutrophils by time of day (hour), median (interquartile range) Related to Figure 2

**Time of Day**	**9 (N=20091)**	**10 (N=28642)**	**11 (N=34487)**	**12 (N=31376)**	**13 (N=31656)**	**14 (N=34257)**	**15 (N=32769)**	**16 (N=34022)**	**17 (N=31456)**	**18 (N=31409)**	**19 (N=13616)**
**CRP**											
mg/litre	2.123 (3.538)	2.173 (3.597)	2.257 (3.838)	2.259 (3.614)	2.312 (3.853)	2.273 (3.826)	2.260 (3.725)	2.245 (3.772)	2.201 (3.778)	2.172 (3.700)	2.140 (3.535)
**WBC**											
(10^9/litre)	5.926 (1.514)	6.046 (1.546)	6.243 (1.564)	6.466 (1.579)	6.713 (1.616)	6.876 (1.622)	6.991 (1.608)	7.078 (1.635)	7.130 (1.616)	7.195 (1.624)	7.254 (1.639)
**Neutrophils**											
(10^9/litre)	3.640 (1.210)	3.717 (1.238)	3.829 (1.241)	3.978 (1.253)	4.159 (1.283)	4.271 (1.279)	4.340 (1.279)	4.339 (1.282)	4.295 (1.258)	4.256 (1.258)	4.231 (1.269)
**Monocytes**											
(10^9/litre)	0.4217 (0.149)	0.4280 (1516)	0.4359 (0.153)	0.4401 (0.1534)	0.4429 (0.1543)	0.4523 (0.1547)	0.4682 (0.1568)	0.4858 (0.1601)	0.4946 (0.1578)	0.5026 (0.1591)	0.5139 (0.1609)
**Lymphocytes**											
(10^9/litre)	1.654 (0.474)	1.697 (0.491)	1.776 (0.516)	1.841 (0.535)	1.904 (0.5525)	1.934 (0.5659)	1.963 (0.5606)	2.027 (0.5777)	2.107 (0.5852)	2.195 (0.6023)	2.263 (0.6292)

Table S5 Diagnostic tests for multicollinearity between predictor variables included in the multiple regression models. Related to Table 2.

Variable	VIF	SQRT VIF	Tolerance	R Squared
Daylength	2.76	1.66	0.3619	0.6381
Sex	1.09	1.04	0.9169	0.0831
Age	1.04	1.02	0.966	0.034
Ethnicity	1.08	1.04	0.922	0.078
Deprivation Index	1.09	1.04	0.9214	0.0786
BMI	1.1	1.05	0.912	0.088
Physical Activity	1.05	1.03	0.9514	0.0486
Sedentary Behaviour	1.09	1.04	0.9163	0.0837
Sleep Duration	1.02	1.01	0.9851	0.0149
Chronotype	1.03	1.01	0.9742	0.0258
Shiftwork	1.05	1.03	0.9489	0.0511
Smoking	1.04	1.02	0.9639	0.0361
Alcohol Frequency	1.04	1.02	0.9657	0.0343
Vitamin D	1.24	1.11	0.8069	0.1931
Outdoor Temperature	3.00	1.73	0.3323	0.6677
Time of Day	1.01	1	0.9932	0.0068
Blood Count Device	1	1	0.9972	0.0028
Assessment Centre	1.04	1.02	0.961	0.039
**Mean VIF**	**1.26**			

Table S6 Multiple regression analysis of associations between time of day and lymphocyte, neutrophil, monocyte counts and CRP. Related to Table 4 and Table 5.

Lymphocytes	Model 1		Model 2		Model 3	
Segment 1	0.051***	[0.050,0.052]	0.050***	[0.049,0.051]	0.050***	[0.049,0.051]
Segment 2	0.082***	[0.079,0.085]	0.085***	[0.082,0.088]	0.085***	[0.082,0.088]
Sex	-0.120***	[-0.124,-0.116]	-0.155***	[-0.159,-0.150]	-0.154***	[-0.158,-0.150]
Age	0.002***	[0.001,0.002]	0.002***	[0.002,0.002]	0.002***	[0.002,0.002]
Ethnicity	0.047***	[0.044,0.049]	0.047***	[0.044,0.051]	0.044***	[0.041,0.047]
Social Deprivation	0.007***	[0.006,0.007]	0.000	[-0.000,0.001]	-0.000	[-0.001,0.000]
BMI			0.022***	[0.021,0.022]	0.022***	[0.021,0.022]
Physical Activity			-0.000***	[-0.000,-0.000]	-0.000***	[-0.000,-0.000]
Sedentary Behaviour			0.009***	[0.008,0.010]	0.008***	[0.007,0.009]
Sleep Duration			0.006***	[0.004,0.008]	0.006***	[0.004,0.008]
Chronotype			-0.015***	[-0.017,-0.012]	-0.015***	[-0.017,-0.012]
Smoking			0.346***	[0.339,0.353]	0.349***	[0.342,0.355]
Alcohol			0.007***	[0.005,0.008]	0.007***	[0.005,0.008]
Daylength					0.000	[-0.000,0.001]
Blood Analyser					-0.005***	[-0.006,-0.003]
Assessment Centre					0.004***	[0.004,0.005]
Observations	309220		275086		272393	
R^2	0.099		0.159		0.161	

95% confidence intervals in brackets
* $p < 0.05$, ** $p < 0.01$, *** $p < 0.001$

Neutrophils	Model 1		Model 2		Model 3	
Segment1	0.130***	[0.127,0.133]	0.126***	[0.123,0.128]	0.125***	[0.122,0.128]
Segment 2	-0.039***	[-0.043,-0.034]	-0.035***	[-0.040,-0.030]	-0.034***	[-0.039,-0.030]
Sex	0.037***	[0.028,0.046]	-0.021***	[-0.031,-0.011]	-0.022***	[-0.031,-0.012]
Age	-0.003***	[-0.003,-0.002]	-0.002***	[-0.003,-0.001]	-0.002***	[-0.003,-0.001]
Ethnicity	-0.153***	[-0.160,-0.147]	-0.146***	[-0.153,-0.139]	-0.151***	[-0.158,-0.144]
Social Deprivation	0.021***	[0.020,0.023]	0.008***	[0.007,0.010]	0.007***	[0.006,0.009]
BMI			0.035***	[0.034,0.036]	0.035***	[0.034,0.036]
Physical Activity			-0.000***	[-0.000,-0.000]	-0.000***	[-0.000,-0.000]
Sedentary Behaviour			0.017***	[0.015,0.019]	0.016***	[0.014,0.018]
Sleep Duration			0.025***	[0.020,0.030]	0.025***	[0.020,0.030]
Chronotype			-0.023***	[-0.028,-0.018]	-0.023***	[-0.028,-0.018]
Smoking			0.794***	[0.779,0.810]	0.797***	[0.781,0.813]
Alcohol			0.023***	[0.020,0.027]	0.023***	[0.020,0.027]
Daylength					-0.016***	[-0.018,-0.015]
Blood Analyser					0.003	[-0.001,0.007]
Assessment Centre					0.007***	[0.006,0.008]
Observations	308545		274483		271793	
R^2	0.042		0.091		0.093	

95% confidence intervals in brackets
* $p < 0.05$, ** $p < 0.01$, *** $p < 0.001$

CRP	Model 1		Model 2		Model 3	
Segment1	0.013*	[0.000,0.026]	0.004	[-0.009,0.017]	0.005	[-0.008,0.019]
Segment 2	-0.010*	[-0.019,-0.002]	-0.013**	[-0.022,-0.005]	-0.013**	[-0.021,-0.004]
Sex	-0.177***	[-0.204,-0.150]	-0.402***	[-0.431,-0.374]	-0.405***	[-0.434,-0.377]
Age	0.028***	[0.026,0.029]	0.025***	[0.024,0.027]	0.026***	[0.024,0.027]
Ethnicity	0.019	[-0.002,0.039]	0.003	[-0.018,0.024]	0.006	[-0.016,0.027]
Social Deprivation	0.054***	[0.050,0.059]	0.027***	[0.022,0.032]	0.029***	[0.024,0.034]
BMI			0.210***	[0.207,0.214]	0.210***	[0.207,0.214]
Physical Activity			-0.001***	[-0.001,-0.001]	-0.001***	[-0.001,-0.001]
Sedentary Behaviour			0.020***	[0.014,0.026]	0.020***	[0.014,0.027]
Sleep Duration			0.045***	[0.031,0.059]	0.045***	[0.031,0.059]
Chronotype			0.029***	[0.014,0.044]	0.029***	[0.014,0.044]
Smoking			0.718***	[0.671,0.765]	0.720***	[0.673,0.767]
Alcohol			0.014**	[0.004,0.024]	0.014**	[0.004,0.024]
Daylength					-0.017***	[-0.022,-0.013]
Assessment Centre					-0.005***	[-0.008,-0.003]
Observations	304000		270518		267872	
R^2	0.006		0.069		0.069	

95% confidence intervals in brackets
* $p < 0.05$, ** $p < 0.01$, *** $p < 0.001$

Monocytes	Model 1		Model 2		Model 3	
Segment1	0.005***	[0.005,0.006]	0.005***	[0.004,0.005]	0.005***	[0.004,0.005]
Segment 2	0.012***	[0.012,0.012]	0.012***	[0.012,0.013]	0.012***	[0.012,0.013]
Sex	0.070***	[0.069,0.071]	0.063***	[0.062,0.064]	0.063***	[0.062,0.064]
Age	0.002***	[0.002,0.002]	0.002***	[0.002,0.002]	0.002***	[0.002,0.002]
Ethnicity	-0.015***	[-0.016,-0.014]	-0.014***	[-0.015,-0.014]	-0.015***	[-0.016,-0.014]
Social Deprivation	0.001***	[0.001,0.001]	0.000**	[0.000,0.001]	0.000	[-0.000,0.000]
BMI			0.004***	[0.004,0.004]	0.004***	[0.004,0.004]
Physical Activity			-0.000***	[-0.000,-0.000]	-0.000***	[-0.000,-0.000]
Sedentary Behaviour			0.001***	[0.001,0.001]	0.001***	[0.001,0.001]
Sleep Duration			0.002***	[0.001,0.003]	0.002***	[0.002,0.003]
Chronotype			-0.000	[-0.001,0.000]	-0.000	[-0.001,0.000]
Smoking			0.050***	[0.048,0.052]	0.050***	[0.048,0.052]
Alcohol			-0.001***	[-0.002,-0.001]	-0.001***	[-0.002,-0.001]
Daylength					0.001***	[0.000,0.001]
Blood Analyser					-0.009***	[-0.010,-0.009]
Assessment Centre					0.001***	[0.001,0.001]
Observations	309207		275076		272380	
R^2	0.091		0.115		0.120	

95% confidence intervals in brackets
* $p < 0.05$, ** $p < 0.01$, *** $p < 0.001$

Table S7 Multiple regression analysis of associations between daylength and lymphocyte, neutrophil, monocyte counts and CRP. Related to Table 2

Neutrophils	Model 1		Model 2		Model 3	
Daylength	0.002***	[0.002,0.003]	0.003***	[0.002,0.004]	0.004***	[0.003,0.005]
Sex	-0.116***	[-0.120,-0.111]	-0.131***	[-0.136,-0.125]	-0.129***	[-0.134,-0.124]
Age	0.001***	[0.001,0.002]	0.002***	[0.001,0.002]	0.001***	[0.001,0.001]
Ethnicity	0.056***	[0.053,0.059]	0.057***	[0.053,0.060]	0.043***	[0.039,0.047]
Social Deprivation	0.007***	[0.006,0.008]	0.001*	[0.000,0.002]	-0.001	[-0.002,0.000]
BMI			0.022***	[0.021,0.023]	0.021***	[0.020,0.022]
Physical Activity			-0.000***	[-0.000,-0.000]	-0.000***	[-0.000,-0.000]
Sedentary Behaviour			0.007***	[0.006,0.009]	0.007***	[0.006,0.008]
Sleep Duration			0.004**	[0.001,0.007]	0.004*	[0.001,0.006]
Chronotype			-0.007***	[-0.009,-0.004]	-0.014***	[-0.017,-0.011]
Shiftwork			-0.018***	[-0.025,-0.011]	-0.009*	[-0.016,-0.002]
Smoking			0.334***	[0.325,0.342]	0.329***	[0.321,0.338]
Alcohol			0.009***	[0.007,0.011]	0.008***	[0.006,0.010]
Vitamin D					-0.000***	[-0.000,-0.000]
Outdoor Temperature					-0.002***	[-0.003,-0.001]
Time of Day					0.057***	[0.057,0.058]
Blood Analyser					-0.005***	[-0.008,-0.003]
Assessment Centre					0.004***	[0.004,0.005]
Observations	308924		175498		161528	
R^2	0.016		0.074		0.175	

95% confidence intervals in brackets
* $p < 0.05$, ** $p < 0.01$, *** $p < 0.001$

**Lymphocytes**	**Model 1**		**Model 2**		**Model 3**	
Daylength	-0.015***	[-0.017,-0.014]	-0.013***	[-0.015,-0.011]	-0.014***	[-0.018,-0.011]
Sex	0.027***	[0.018,0.036]	-0.121***	[-0.133,-0.108]	-0.127***	[-0.140,-0.115]
Age	-0.000	[-0.001,0.000]	-0.006***	[-0.007,-0.005]	-0.007***	[-0.008,-0.006]
Ethnicity	-0.141***	[-0.148,-0.134]	-0.139***	[-0.148,-0.131]	-0.168***	[-0.178,-0.159]
Social Deprivation	0.023***	[0.021,0.024]	0.005***	[0.003,0.007]	0.001	[-0.001,0.003]
BMI			0.039***	[0.038,0.040]	0.037***	[0.035,0.038]
Physical Activity			-0.000	[-0.000,0.000]	-0.000	[-0.000,0.000]
Sedentary Behaviour			0.018***	[0.015,0.020]	0.018***	[0.015,0.021]
Sleep Duration			0.023***	[0.017,0.029]	0.023***	[0.017,0.030]
Chronotype			-0.008*	[-0.015,-0.002]	-0.018***	[-0.025,-0.012]
Shiftwork			0.015	[-0.001,0.032]	0.031***	[0.015,0.048]
Smoking			0.773***	[0.754,0.793]	0.760***	[0.740,0.780]
Alcohol			0.020***	[0.015,0.024]	0.018***	[0.014,0.023]
Vitamin D					-0.003***	[-0.003,-0.002]
Outdoor Temperature					0.002	[-0.000,0.004]
Time of Day					0.070***	[0.068,0.072]
Blood Analyser					0.000	[-0.005,0.006]
Assessment Centre					0.007***	[0.006,0.008]
Observations	308250		175049		161112	
R^2	0.008		0.062		0.093	

95% confidence intervals in brackets
* $p < 0.05$, ** $p < 0.01$, *** $p < 0.001$

**Monocytes**	**Model 1**		**Model 2**		**Model 3**	
Daylength	0.001***	[0.001,0.001]	0.001***	[0.001,0.001]	-0.000*	[-0.001,-0.000]
Sex	0.071***	[0.070,0.072]	0.058***	[0.056,0.059]	0.057***	[0.056,0.059]
Age	0.002***	[0.002,0.002]	0.001***	[0.001,0.001]	0.001***	[0.001,0.001]
Ethnicity	-0.013***	[-0.014,-0.013]	-0.013***	[-0.014,-0.012]	-0.016***	[-0.017,-0.015]
Social Deprivation	0.001***	[0.001,0.002]	0.000	[-0.000,0.000]	-0.000	[-0.000,0.000]
BMI			0.004***	[0.004,0.004]	0.004***	[0.004,0.004]
Physical Activity			-0.000	[-0.000,0.000]	-0.000	[-0.000,0.000]
Sedentary Behaviour			0.001***	[0.001,0.002]	0.001***	[0.001,0.002]
Sleep Duration			0.002***	[0.001,0.003]	0.002***	[0.001,0.002]
Chronotype			0.001*	[0.000,0.002]	-0.001	[-0.001,0.000]
Shiftwork			0.000	[-0.002,0.002]	0.001	[-0.000,0.003]
Smoking			0.048***	[0.046,0.050]	0.048***	[0.045,0.050]
Alcohol			-0.001***	[-0.002,-0.001]	-0.002***	[-0.002,-0.001]
Vitamin D					-0.000***	[-0.000,-0.000]
Outdoor Temperature					0.001***	[0.001,0.001]
Time of Day					0.010***	[0.009,0.010]
Blood Analyser					-0.010***	[-0.010,-0.009]
Assessment Centre					0.001***	[0.001,0.001]
Observations	308911		175441		161471	
R^2	0.060		0.075		0.119	

95% confidence intervals in brackets
* $p < 0.05$, ** $p < 0.01$, *** $p < 0.001$

CRP	Model 1		Model 2		Model 3	
Daylength	-0.006***	[-0.007,-0.004]	-0.005***	[-0.006,-0.003]	-0.004***	[-0.007,-0.002]
Sex	-0.042***	[-0.050,-0.035]	-0.153***	[-0.162,-0.144]	-0.085***	[-0.108,-0.063]
Age	0.016***	[0.016,0.017]	0.012***	[0.012,0.013]	0.012***	[0.011,0.013]
Ethnicity	0.005	[-0.000,0.011]	-0.001	[-0.007,0.006]	-0.003	[-0.010,0.004]
Social Deprivation	0.020***	[0.019,0.022]	0.005***	[0.003,0.006]	0.005***	[0.003,0.007]
BMI			0.104***	[0.103,0.105]	0.100***	[0.097,0.102]
Physical Activity			-0.000***	[-0.000,-0.000]	-0.000***	[-0.000,-0.000]
Sedentary Behaviour			0.014***	[0.012,0.016]	0.014***	[0.012,0.016]
Sleep Duration			0.017***	[0.012,0.022]	0.016***	[0.011,0.021]
Chronotype			0.015***	[0.010,0.020]	0.016***	[0.011,0.021]
Shiftwork			0.050***	[0.038,0.062]	0.048***	[0.036,0.061]
Smoking			0.282***	[0.268,0.297]	0.281***	[0.267,0.296]
Alcohol			0.012***	[0.009,0.015]	0.012***	[0.009,0.016]
Vitamin D					-0.002**	[-0.004,-0.001]
Sex					0.000	[0.000,0.000]
Vitamin D # Sex					-0.001***	[-0.002,-0.001]
Vitamin D					0.000	[0.000,0.000]
BMI					0.000	[0.000,0.000]
Vitamin D # BMI					0.000***	[0.000,0.000]
Outdoor Temperature					-0.000	[-0.002,0.002]
Time of Day					-0.004***	[-0.005,-0.002]
Blood Analyser					-0.000	[-0.004,0.004]
Assessment Centre					-0.001**	[-0.002,-0.000]
Observations	303707		172476		161492	
R^2	0.019		0.223		0.222	

95% confidence intervals in brackets
* $p < 0.05$, ** $p < 0.01$, *** $p < 0.001$

WBC	Model 1		Model 2		Model 3	
Daylength	-0.011***	[-0.013,-0.009]	-0.008***	[-0.011,-0.006]	-0.011***	[-0.015,-0.007]
Sex	0.005	[-0.007,0.016]	-0.181***	[-0.197,-0.166]	-0.186***	[-0.202,-0.171]
Age	0.002***	[0.002,0.003]	-0.003***	[-0.004,-0.002]	-0.005***	[-0.006,-0.004]
Ethnicity	-0.098***	[-0.107,-0.090]	-0.094***	[-0.105,-0.083]	-0.142***	[-0.153,-0.130]
Social Deprivation	0.033***	[0.031,0.035]	0.006***	[0.004,0.009]	0.001	[-0.002,0.003]
BMI			0.068***	[0.066,0.069]	0.064***	[0.062,0.066]
Physical Activity			-0.000*	[-0.000,-0.000]	-0.000**	[-0.000,-0.000]
Sedentary Behaviour			0.027***	[0.023,0.030]	0.027***	[0.024,0.030]
Sleep Duration			0.031***	[0.023,0.039]	0.030***	[0.022,0.038]
Chronotype			-0.013**	[-0.022,-0.005]	-0.033***	[-0.041,-0.025]
Shiftwork			-0.000	[-0.021,0.020]	0.027*	[0.006,0.047]
Smoking			1.223***	[1.198,1.248]	1.205***	[1.180,1.230]
Alcohol			0.028***	[0.022,0.033]	0.025***	[0.019,0.031]
Vitamin D					-0.003***	[-0.004,-0.003]
Outdoor Temperature					0.001	[-0.001,0.004]
Time of Day					0.142***	[0.139,0.144]
Blood Analyser					-0.016***	[-0.023,-0.009]
Assessment Centre					0.012***	[0.011,0.014]
Observations	309516		175753		161746	
R^2	0.005		0.089		0.163	

95% confidence intervals in brackets
* $p < 0.05$, ** $p < 0.01$, *** $p < 0.001$

Table S8 Comparison of demographic and lifestyle characteristics of participants that self-reported good health and were included in this study, and the remainder of the UK Biobank database, that self-reported poor health. Related to Methods section and Table 1

	**Healthy** (N=329261)	**Diseased** (N=159904)
**Age (years)**		
Mean (SD)	55 (± 8)	58 (± 8)
**Sex**		
Female	185438 (56 %)	80199 (50 %)
Male	143823 (44 %)	79705 (50 %)
**Social Deprivation Index**		
Mean (SD)	-1.544 (± 2.945)	-0.8489 (± 3.296)
**Ethnicity**		
White	311054 (94 %)	150657 (94 %)
Asian	6369 (2 %)	3022 (2 %)
Black	4902 (1 %)	2817 (2 %)
Chinese	1190 (0 %)	302 (0 %)
Mixed	1929 (1 %)	931 (1 %)
Other	2849 (1 %)	1491 (1 %)
**Physical Activity**		
Mean (SD)	45.89 (± 62.72)	40.63 (± 62.25)
**Sedentary Behaviour**		
Mean (SD)	4.90 (± 2.24)	5.31 (± 2.50)
**BMI**		
Mean (SD)	26.82 (± 4.34)	28.64 (± 5.39)
**Smoker**		
No	296633 (90 %)	139513 (87 %)
Yes	31606 (10 %)	19615 (12 %)
**Chronotype**		
Evening	23586 (7 %)	15512 (10 %)
More evening than morning	82570 (25 %)	40817 (26 %)
More morning than evening	107374 (33 %)	46633 (29 %)
Morning	79704 (24 %)	38064 (24 %)

Table S9 Comparison of demographic and lifestyle characteristics of participants that were randomly selected by UK Biobank for inclusion in the study of blood levels of antibodies against 20 infectious agents, and the remainder of the UK Biobank database. Related to Table 1

	**Not Antigen Tested** (N=493224)	**Antigen Tested*** (N=9431)
**Age (years)**		
Mean (SD)	56 (± 8)	56 (± 8)
**Sex**		
Female	268121 (54 %)	5277 (56 %)
Male	224972 (46 %)	4153 (44 %)
**Social Deprivation Index**		
Mean (SD)	-1.292 (± 3.096)	-1.368 (± 3.052)
**Ethnicity**		
White	463829 (94 %)	8883 (94 %)
Asian	9687 (2 %)	195 (2 %)
Black	7920 (2 %)	141 (1 %)
Chinese	1538 (0 %)	36 (0 %)
Mixed	2905 (1 %)	53 (1 %)
Other	4481 (1 %)	78 (1 %)
**Physical Activity**		
Mean (SD)	43.94 (± 62.65)	43.85 (± 58.91)
**Sedentary Behaviour**		
Mean (SD)	5.02 (± 2.35)	5.01 (± 2.28)
**BMI**		
Mean (SD)	27.43 (± 4.80)	27.33 (± 4.80)
**Smoker**		
No	438167 (89 %)	8429 (89 %)
Yes	52028 (11 %)	949 (10 %)
**Chronotype**		
Evening	39375 (8 %)	736 (8 %)
More evening than morning	123899 (25 %)	2435 (26 %)
More morning than evening	154370 (31 %)	3009 (32 %)
Morning	118100 (24 %)	2271 (24 %)

*Participants randomly selected for inclusion in serum antigen titre study

Transparent Methods

Study Sample

The study sample were participants of UK Biobank, a general population cohort study that recruited over half a million UK residents continuously between 2006 and 2010, at 22 assessment centres located across the UK (www.ukbiobank.co.uk). Eligible participants who lived within travelling distance of one of 22 UK assessment centres were identified through national health service patient registers and invited to participate by mail, resulting in a 5.5% response rate (Sudlow et al., 2015). The participants were aged between 37-73 years at the time of enrolment. Inclusion in the present study was restricted to participants who reported having no chronic disease at the time of recruitment. Participants provided full informed consent to participate in UK Biobank. This study was covered by the generic ethical approval for UK Biobank studies from the NHS National Research Ethics Service

Participant Measures

Participants were invited to attend the assessment centre at a pre-booked provisional appointment time between 8am-7pm; they did not self-select the time of day of attendance but were free to reschedule if required. Baseline information was collected at the assessment centre using a questionnaire and an interview, and blood samples and physical measurements were taken. Information on the demographic status of the participants included age at baseline, sex (male/female), ethnicity (White, Black, Mixed, Chinese, Asian, Other).(large-scale and resource, 2007) Participants were self-categorised as morning or evening chronotype using the question, "*Do you consider yourself to be: Definitely a morning person; More a morning than evening person; More an evening than a morning person; Definitely an evening person; or Don't know".* The self-reported level and duration of usual physical activity was used to derive total physical activity, measured as metabolic equivalents (MET.hours/week). A proxy of sedentary behaviour was derived from the total number of self-reported hours spent driving, using a computer and watching television each day. Smoking status was self-reported and categorized as "*never smoker*," "*current smoker*" and "*former smoker*." The frequency and volume of alcohol intake were self-reported. Body mass index (BMI was measured by trained UK Biobank staff using standardized methods and instruments. Habitual sleep duration was self-reported in hours per 24h.(large-scale and resource, 2007)

Blood samples were collected at the end of the assessment centre visit, and the time was immediately recorded on computerised system by swiping the unique barcode on the collection tube. Blood cell counts were performed within 24 hours using an LH750 haematology analyser (Coulter, Beckman Coulter, Brea, CA, USA) to determine the total number of white blood cells, plus the numbers of neutrophils, lymphocytes and monocytes, which were expressed as a percentage of the total white blood cell count. Vitamin D was measured in serum using a chemiluminescent direct competitive immunoassay (LIAISON XL; Diasorin, Italy). Serum C reactive protein (CRP) was measured using a high-sensitivity immunoturbidimetric assay

performed on a Beckman Coulter clinical chemistry analyser (AU5800 Immuno-turbidimetric, Beckman Coulter (UK)).

A subset of participants (n=9724) were chosen at random for assessment of blood levels of antibodies against 20 infectious agents (see tables S7-8 for details of antigens). Antibody levels were measured using a Luminex high-throughput platform following validation against gold-standard assays and independent reference sera. Results of analyses were expressed as median fluoresce intensity for each antigen, and seropositivity status based on suggested thresholds. Full details of the participant measures and analysis procedures are available at www.ukbiobank.co.uk.

Environmental Variables

Latitude and longitude were derived from the postcode of residence at 1km^2 resolution using Open Source Geographic Information System software (QGIS Open Source Geospatial Foundation Project, http://qgis.osgeo.org). These data were combined with information on the date of attendance at the assessment center to derive the length of daylight on that day for each participant using vectorial algorithms in R-software [R version] in the '*insol*' package (*insol:* Solar Radiation). Daylight was approximated over the hours (9am-7pm) of the diurnal dataset by calculating the mean zenith angle of the sun at each time and assessment centre location using the R-package "*geolight*".(Lisovski and Hahn, 2012) The derived data were verified using information provided by the Global Monitoring Division of the US Government National Oceanic and Atmospheric Administration. Outdoor temperature was averaged for the 3 weeks preceding the date of attendance from data provided by the UK Meteorological Office for the weather station nearest to each assessment centre. Monthly data was aggregated into seasons for the purposes of descriptive analysis with seasons defined as: Spring = February, March, April; Summer = May, June, July; Autumn = August, September, October; Winter = November, December, January.

Data Analysis

Seasonal and daily variation were assessed by plotting mean values of white blood cell and CRP values against month or hour of sample collection, fitting models to describe annual and daily variation, and then investigating whether any variation was independent of confounding factors and directly related to day length. The sampling distribution for CRP was positively-skewed, and a logarithmic transformation was applied before regression analysis, but original data are shown in the descriptive data summaries.

Seasonal patterns were analyzed by fitting a linear regression model for each outcome of interest that included a sine and a cosine term of transformations of the time variable, taken as month:

$$Y_i = M + \beta \, Cos\,(2\pi \, t_i /12) + \gamma \, Sin\,(2\pi \, t_i /12) \quad (1)$$

Where Y is t is time (months), and M, β and γ were predicted by regression, above. The acrophase (Φ) and amplitude (A) was predicted using equations 2 and 3, with M predicted from equation (1) above.

$$A = (\beta^2 + \gamma^2)^{\frac{1}{2}} \quad (2)$$

$$\Phi = \tan^{-1}(-\gamma/\beta) \quad (3)$$

The intercept (M) was the mean level of the curve and thus an estimate of the annual mean of each outcome variable. The amplitude (A) was the distance from the mean to the acrophase or the nadir, providing an estimate of the magnitude of seasonality. The acrophase (Φ) is the peak x axis value of the curve, whereas the nadir is the trough. Seasonality was indicated by statistical significance of the estimated cosinor (sine and cosine) regression coefficients.

Variation of the markers over the daily time course of sample collection was modeled using linear methods since the absence of nighttime samples precluded assumption of circadian patterns. Although assessment centre appointments started at 8am, the blood sample was collected at the end of the 40-minute assessment, so the 8am time point was excluded due to small sample numbers at this time (n= 1843). The relationship between time of day and the immune parameters was represented by a series of linear regression lines connected at breakpoints where the slope of each line changed. This analysis was implemented using the R package "segmented" to predict the times of breakpoints during the test period for each analyte (Muggeo, 2003). The statistical significance of the segmented regression model was assessed using the Davies test to test the null hypothesis that a breakpoint does not exist, and that the difference in slope parameter (ψ) of the segmented relationship is zero. The breakpoints and slopes of each segment indicate peaks and troughs in WBC and CRP levels over time, as well as the rate and direction of any changes.

If seasonal and daily variation were indicated, we next investigated if these patterns were related to day length, and to time of day, and if any relationships were independent of lifestyle and environmental factors. The daytime data were modelled as a series of linear splines to account for the non-linear relationships between time of day and the immune parameters. Three multiple linear regression models were run that included an increasing number of covariates and progressively adjusted for sociodemographic, disease, lifestyle and environmental (temperature and day length) factors, with results reported as point estimates and 95% confidence intervals.

Potential confounders included as covariables were age; sex; ethnicity; Townsend area-deprivation score; physical activity and sedentary behaviour; alcohol intake and smoking status; outdoor temperature; blood analyser; vitamin D; sleep duration; chronotype and UK Biobank assessment centre. Multicollinearity between the covariables was assessed using variance inflation factors (VIF) and tolerance factors, with values of VIF > 10 taken to denote problematic collinearity.

All analyses were performed using R version 3.5, and Stata 14 statistical software (StataCorp LP) and values of $p < 0.01$ were considered to represent statistical significance.

Supplemental References

Lisovski, S., Hahn, S., 2012. GeoLight - processing and analysing light-based geolocator data in R. Methods Ecol. Evol. 3, 1055-1059

Muggeo, V.M.R., 2003. Estimating regression models with unknown break-points. Stat. Med. 22, 3055–3071.

Sudlow, C., Gallacher, J., Allen, N., Beral, V., Burton, P., Danesh, J., Downey, P., Elliott, P., Green, J., Landray, M., Liu, B., Matthews, P., Ong, G., Pell, J., Silman, A., Young, A., Sprosen, T., Peakman, T., Collins, R., 2015. UK biobank: an open access resource for identifying the causes of a wide range of complex diseases of middle and old age. PLoS Med 12, e1001779.
